# Association of post-diagnostic use of cholera vaccine with survival outcome in breast cancer patients

**DOI:** 10.1038/s41416-020-01108-9

**Published:** 2020-10-07

**Authors:** Guoqiao Zheng, Jan Sundquist, Kristina Sundquist, Jianguang Ji

**Affiliations:** 1 Center for Primary Health Care Research, Lund University/Region Skåne, Malmö, Sweden; 2 Department of Family Medicine and Community Health, Department of Population Health Science and Policy, Icahn School of Medicine at Mount Sinai, New York, NY USA; 3 Center for Community-based Healthcare Research and Education (CoHRE), Department of Functional Pathology, School of Medicine, Shimane University, Shimane, Japan

**Keywords:** Breast cancer, Cancer epidemiology

## Abstract

**Background:**

Expensive cancer treatment calls for alternative ways such as drug repurposing to develop effective drugs. The aim of this study was to analyse the effect of post-diagnostic use of cholera vaccine on survival outcome in breast cancer patients.

**Methods:**

Cancer diagnosis and cholera vaccination were obtained by linkage of several Swedish national registries. One vaccinated patient was matched with maximum two unvaccinated individuals based on demographic, clinical and socioeconomic factors. We performed proportional Cox regression model to analyse the differences in overall and disease-specific survivals between the matched patients.

**Results:**

In total, 617 patients received cholera vaccine after breast cancer diagnosis. The median (interquartile range) time from diagnosis to vaccination was 30 (15–51) months and from vaccination to the end of follow-up it was 62 (47–85) months. Among them, 603 patients were matched with 1194 unvaccinated patients. Vaccinated patients showed favourable overall survival (hazard ratio (HR): 0.54, 95% confidence interval (CI): 0.37–0.79) and disease-specific survival (HR: 0.53, 95% CI: 0.33–0.84), compared to their unvaccinated counterpart. The results were still significant in multiple sensitivity analyses.

**Conclusions:**

Post-diagnostic use of cholera vaccine is associated with a favourable survival rate in breast cancer patients; this provides evidence for repurposing it against breast cancer.

## Background

Breast cancer is the most common cancer found among women worldwide. Although the survival of breast cancer is increasing with the advancement of treatment, it is still the leading cause of death due to cancer among women.^1^ The development of targeted therapy on breast cancer is both time-consuming and expensive. It is estimated that a typical drug development usually takes 15–18 years and costs approximately 2–3 billion dollars.^2^ In a clinical setting, cancer patients and their involved family members suffer from the pressure of meeting the costs of these expensive cancer drugs financially as well as the emotional burden associated with the treatment. Some of these expensive cancer drugs are not covered by the public healthcare system in many developing countries thus leading to a higher mortality rate among insolvent patients with breast cancer.^3^ In this scenario, drug repurposing is an alternative and efficient way for drug development, which identifies the new indication of the drug outside the scope of the original medical condition. For example, raloxifene, which was originally used to treat osteoporosis, was approved by the U.S Food and Drug Administration for invasive breast cancer treatment in 2007.^4^

Cholera vaccine is widely used among people travelling to regions with a high prevalence of cholera infection. Cholera toxin is composed of two subunits: the A subunit (CTA) and the B subunit (CTB). The functional component of the vaccine is CTA. Many studies have shown that cholera toxin can suppress the proliferation of several cancer cell lines, including breast cancer, by inhibiting growth factor signal transduction pathway or by triggering apoptosis.^5^ Cholera toxin has been reported to have immunomodulatory properties.^6–9^ In vitro experiments have shown that recombinant CTB can activate dendritic cells and enhance antitumour immunity.^6^ Cholera toxin suppressed carcinogenesis in a mouse model of inflammation-driven sporadic colon cancer.^10^ Recently, post-diagnostic use of cholera vaccine has been shown to be of benefit in disease-specific survival of colorectal and prostate cancers.^11,12^ The aim of this study was to evaluate whether the antitumour effect of cholera vaccine could be valid in breast cancer patients by analysing data derived from several Swedish national registries. To the best of our knowledge, this is the first national population-based cohort study on the association of post-diagnostic use of cholera vaccine and breast cancer survival, which may provide new evidence for breast cancer treatment.

## Methods

**Fig. 1:**
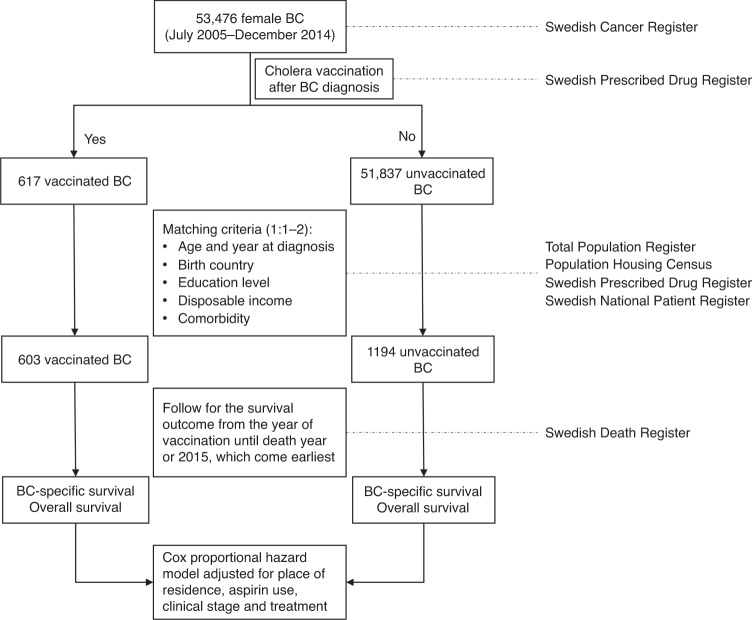
Flowchart of the study. BC breast cancer.

This study was performed based on the linkage of several national Swedish registries and how the study was performed is shown in Fig. 1. Female patients, who were diagnosed with primary invasive breast cancer, were identified from the Swedish Cancer Registry by using the Tenth Version of International Classification of Disease (ICD-10) code of C50. The clinical stage of breast cancer at diagnosis was classified into four groups (stage I, stage II, stage II and stage IV) based on the tumour size (T), nodal status (N) and the presence of metastasis (M) according to the seventh edition of the American Joint Committee on Cancer staging manual.^13^ The TNM staging system has been used in the cancer registry since 2003.

Data on post-diagnostic use of cholera vaccine were extracted from the Swedish Prescribed Drug Register. As this register was established in July 2005 and was updated until December 2014, breast cancer patients diagnosed during only this period were included in the study. The Anatomical Therapeutic Chemical (ATC) Classification System was applied in the drug register and the administration of cholera vaccine was identified by code “J07AE01”. The ATC code for aspirin use was B01AC06, which was also considered in our analysis, as aspirin use in breast cancer patients was associated with decreased mortality.^14^ As the information on hormone receptor status was not available, we used medical treatments as a proxy for the identification of hormone receptor status, which included treatment with anti-oestrogens (L02BA), aromatase inhibitors (L02BG) and gonadotropin-releasing hormone analogues (L02AE).

The date of death, as well as the underlying cause of death during the study period, was obtained from the Swedish Death Register. The primary outcome was death due to breast cancer (ICD-10 code: C50) and the secondary outcome was death due to all causes (ICD-10 code: A00 to Z99).

Patients’ demographic and socioeconomic factors including country of birth (Sweden, other European countries and non-European countries), educational level (1–9 years, 10–11 years and ≥12 years of education), disposable income (lowest, middle–low, middle–high, highest) and place of residence (big cites, other southern and northern cities) at diagnosis were obtained from the Total Population Register and the Population Housing Census. Comorbidity at the diagnosis of breast cancer was extracted from the Swedish National Patient Register and the diseases for the calculation of Charlson Comorbidity Index were considered.^15^

A total of 52,454 breast cancer patients were diagnosed between July 2005 and December 2014, among which 617 had post-diagnostic use of cholera vaccine. The characteristics of patients stratified by cholera vaccination are shown in Supplementary Table 1. Considering the possibility that patients using cholera vaccine might be healthier or associated with better socioeconomic status, we matched each vaccinated patient with at most two patients who did not receive the vaccine. The matching conditions included year of diagnosis, age at diagnosis (5-year gap), education level, comorbidity (yes or no), disposable income and country of birth. Pearson’s Chi-square tests, or Fisher Exact tests when appropriate, were performed to compare the difference of these characteristics between the two groups. The follow-up commenced from the date of administration of cholera vaccine for the vaccinated patients. For the unvaccinated patients, it commenced from the date of vaccination matched in each stratum. The follow-up was terminated in the year of death or 2015, whichever came earliest. Cox proportional hazard regression model was used to analyse the effect of post-diagnostic use of cholera vaccine on all-cause and disease-specific survival with further adjustment of clinical stage, aspirin use, place of residence and hormone therapy. Kaplan–Meier plot was generated for disease-specific survival since the cholera vaccination.

To avoid chance findings, several sensitivity analyses were performed. The effect of competing risks as a result of death from other causes was analysed by using the sub-distribution hazards model proposed by Fine and Gray.^16^ The exposure of cholera vaccine was considered with 1-year lag given that short duration of exposure is unlikely to be associated with the mortality outcome. As they were able to travel abroad, patients who received cholera vaccine could have been healthier and associated with better socioeconomic status compared to their non-receiving counterparts. To avoid the indication bias, effects of post-diagnostic use of antimalarial medication on the breast cancer survival were analysed by using the same matching approach. In Sweden, malarone (atovaquone/proguanil) (ATC code: P01BB51), mefloquine (ATC code: P01BA05 and P01BC02) and doxycycline are usually recommended for the prevention of malaria. However, doxycycline is normally used for the treatment of bacterial infection, thus it is not suitable to be included in this study.^17^ In addition, influence of use of cholera vaccine before breast cancer diagnosis on the survival rate was evaluated. Finally, we performed sensitivity analyses by excluding patients with advanced breast cancer (clinical stages of III and IV) and by including patients with hormone therapy.

All the statistical analyses were performed in SAS environment (version 9.3). The survival curve was generated in R (version 3.3.5). Statistical comparisons were two tailed and *P* value < 0.05 was considered statistically significant.

## Results

Among the 617 breast cancer patients with post-diagnostic use of cholera vaccine, the median (interquartile range (IQR)) time from breast cancer diagnosis to vaccination was 30 (15–51) months, and the median (IQR) time from vaccination to the end of follow-up was 62 (47–85) months. The median age at diagnosis of breast cancer was 64 years. In the matched setting, 603 vaccinated patients were able to match with 1194 unvaccinated individuals. The demographic, clinical and socioeconomic characteristics of the two groups are displayed in Table 1. Age at diagnosis, year of diagnosis, birth country, education level, disposable income and comorbidity were found to be well distributed based on Pearson’s Chi-square test. As for the unmatched factors, no significant difference was found for place of residence, use of aspirin and clinical stage. In the final regression model, these unmatched factors were adjusted. Most of the patients were born in Sweden (92%) and diagnosed before the age of 65 years (80%). Approximately half of them had >11 years of education (54%), had the highest disposable income (41%) and were living in big cities (53%). Nearly 15% of them had a history of aspirin use and 14% had comorbidity upon diagnosis. More than half of them were diagnosed with stage II breast cancer.

**Table 1:** Characteristics of matched breast cancer patients diagnosed from 2005 to 2014.

Characteristics	No use		Cholera vaccine use		*P* value for Chi-square test
	*N*	%	*N*	%	
Age at diagnosis (years)					
≤65	954	79.9	482	79.9	0.9864
>65	240	20.1	121	20.1	
Year of diagnosis					
2005–2010	944	79.1	477	79.1	0.9833
2011–2014	250	20.9	126	20.9	
Birth country					
Sweden	1105	92.6	556	92.2	0.9651
Other European countries	78	6.5	41	6.8	
Non-European countries	11	0.9	6	1.0	
Education level, years					
1–9	99	8.3	51	8.5	0.9912
10–11	440	36.8	221	36.6	
≥12	655	54.9	331	54.9	
Disposable income					
Lowest	149	12.5	76	12.6	0.9456
Middle–low	230	19.3	115	19.1	
Middle–high	323	27.0	162	26.9	
Highest	489	41.0	247	41.0	
Missing value	3	0.2	3	0.5	
Place of residence					
Big cites	630	52.8	321	53.2	0.7158
Southern Sweden	338	28.3	177	29.4	
Northern Sweden	226	18.9	105	17.4	
Comorbidity^a^					
No	1031	86.4	519	86.1	0.8713
Yes	163	13.6	84	13.9	
Aspirin use					
No	993	83.2	512	84.9	0.1205
Yes	201	16.8	91	15.1	
Clinical stage					
I	413	33.1	239	39.6	0.1538^b^
I	750	62.8	352	57.4	
III	24	2.0	8	1.3	
IV	7	0.6	4	0.7	
Hormone therapy^c^					
No	281	23.5	147	24.4	0.6917
Yes	913	76.5	456	75.6	
Total	1194	100	603	100	

^a^Diseases for the calculation of Charlson Comorbidity Index considered: myocardial infarction, congestive heart failure, peripheral vascular disease, cerebrovascular disease, dementia, chronic pulmonary disease, rheumatic disease, peptic ulcer disease, mild liver disease, diabetes, hemiplegia or paraplegia, renal disease, any malignancy (including lymphoma and leukaemia, except malignant neoplasm of skin), moderate-to-severe liver disease, metastatic solid tumour and AIDS/HIV.^15^

^b^Fisher Exact test was performed. Matching variables included year of diagnosis, age at diagnosis (5-year gap), education level, comorbidity (yes or no), income and birth country. One vaccinated patient was matched with at most two patients without vaccine use. In the final regression model, place of residence, use of aspirin and clinical stage were adjusted.

^c^Hormone therapy included anti-oestrogens (ATC, L02BA), aromatase inhibitors (L02BG) and gonadotropin-releasing hormone analogues (L02AE).

**Fig. 2:**
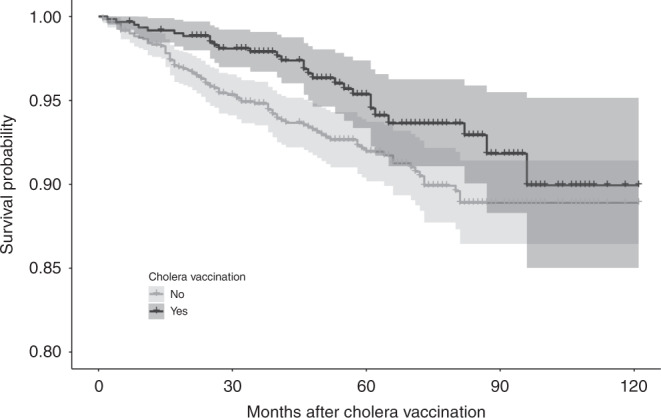
Kaplan–Meier plot for disease-specific survival stratified by cholera vaccination. The area within the band is the confidence interval of the survival probability.

The Kaplan–Meier survival curve in Fig. 2 shows that the disease-specific survival in patients with cholera vaccination was better than those without. After 5 years of cholera vaccination, the disease-specific survival (95% confidence interval (CI)) was 95.3% (93.4–97.4%) for patients with vaccination and 91.9% (90.2–93.7%) for those without. After 10 years, the survival rate (95% CI) was 94.1% (91.8–96.5%) and 89.9% (88.0–91.9%), respectively. Table 2 displays the effects of post-diagnostic use of cholera vaccine on overall and disease-specific survival in the matched breast cancer patients. After the respective median (IQR) follow-up time of 62 (47–85) and 62 (45–85) months, 39 vaccinated and 127 unvaccinated patients died, thus resulting in a better overall survival for patients with vaccine (hazard ratio (HR): 0.54, 95% CI: 0.37–0.79). Considering that death was only caused by breast cancer, the difference in survival probability was significant (HR: 0.53, 95% CI: 0.33–0.84).

**Table 2:** Effects of post-diagnostic use of cholera vaccine on breast cancer survival.

Groups	No. of persons at risk	No. of deaths	HR (95% CI)	*P* value
Overall survival				
No cholera vaccine	1194	127	–	–
Cholera vaccine use	603	39	0.54 (0.37–0.79)	0.0017
Disease-specific survival				
No cholera vaccine	1194	90	–	–
Cholera vaccine use	603	28	0.53 (0.33–0.84)	0.0070

Table 3 displays the results from the sensitivity analyses. While considering the effect of competing risks from other cause of death, the vaccinated patients still experienced better survival compared to their unvaccinated counterparts (HR: 0.55, 95% CI: 0.37–0.81). By defining the exposure period as 1 year after the cholera vaccine administration, similar sets of analyses were performed for overall (HR: 0.57, 95% CI: 0.38–0.88) and disease-specific survival (HR: 0.56, 95% CI: 0.33–0.95). A total of 1013 patients were vaccinated before their breast cancer diagnosis. After applying the same approach, cholera vaccination before breast cancer diagnosis did not show a significant effect on the disease-specific survival (HR: 1.04, 95% CI: 0.66–1.64). When the analysis included only patients with clinical stages of I and II breast cancer, the result was still significant (HR: 0.59, 95% CI: 0.37–0.94). Among individuals with hormone therapy, the protective nature of the vaccination showed borderline significance (HR: 0.60, 95% CI: 0.34–1.04).

**Table 3:** Sensitivity analyses.

Category	No. of persons at risk	No. of deaths	HR (95% CI)	*P* value
Disease-specific survival considering competing event				
No cholera vaccine	1194	90	–	–
Cholera vaccine use	603	28	0.55 (0.37–0.81)	0.0026
Overall survival after 1 year				
No cholera vaccine	1180	104	–	–
Cholera vaccine use	597	33	0.57 (0.38–0.88)	0.0101
Disease-specific survival after 1 year				
No cholera vaccine	1180	72	–	–
Cholera vaccine use	597	23	0.56 (0.33–0.95)	0.0323
Disease-specific survival regarding cholera vaccine use before breast cancer diagnosis^a^				
No cholera vaccine	1983	123	–	–
Cholera vaccine use	998	45	1.04 (0.66–1.64)	0.8751
Disease-specific survival after antimalarial medication^b^				
No antimalarial medication	873	48	–	–
Antimalarial medication	444	18	1.14 (0.57–2.29)	0.7141
Disease-specific survival in patients with stages I and II				
No cholera vaccine	1166	78		
Cholera vaccine use	589	26	0.59 (0.41–0.91)	0.0271
Disease-specific survival in patients with hormone therapy				
No cholera vaccine	892	56		
Cholera vaccine use	452	20	0.60 (0.34–1.04)	0.0711

^a^Number of breast cancer patients with pre-diagnostic use of cholera vaccine was 1013. Similar matching conditions were performed and 998 exposed patients were paired with 1983 unexposed patients.

^b^Number of breast cancer patients with post-diagnostic use of antimalarial medication was 598, among which 468 were without cholera vaccine administration. Similar matching conditions were performed for the exposed patients. In the final matched setting, there were 444 patients exposed to post-diagnostic use of antimalarial treatment as well as 873 patients without.

Next, the effect (if any) of antimalarial medication was assessed to account for chance findings due to indication bias. Notably, 598 patients had post-diagnostic antimalarial medication, and 130 of them had previously used cholera vaccine. To remove the protective effects of cholera vaccine, 468 unvaccinated patients were retained. After matching 444 patients with 873 individuals without antimalarial medication, we found that antimalarial medication was not significantly associated with disease-specific survival (HR: 1.14, 95% CI: 0.57–2.29).

## Discussion

With better understanding of cancer biology and more advanced technology, various antitumour drugs have been developed to fight against cancer. However, the process from drug discovery to the ultimate approval for clinical application is usually lengthy and costly with an accompanying low success rate. Drug repurposing for oncology that studies the antitumour effects for drugs available for other diseases is relatively cheaper and faster than the classical drug discovery process as the safety and toxicity of the drugs are already known.^18^ The aim of the current study was therefore to serve the drug repurposing approach for breast cancer. To our best knowledge, it is the first nationwide population-based study evaluating the association between post-diagnostic use of cholera vaccine and disease-specific survival in breast cancer. Consistent with the results reported for colorectal and prostate cancer,^11,12^ vaccinated breast cancer patients were observed with 47% decreased hazard from breast cancer compared to the unvaccinated individuals. The results remained significant in various sensitivity analyses.

When estimating the effects of medication use on health outcomes, many issues should be considered, such as immortal time bias, indication bias, confounding, etc. In order to control immortal time bias, we started the follow-up from the administration of cholera vaccination. Compared to breast cancer patients without cholera vaccination (Supplementary Table 1), those who had been vaccinated tended to be younger, diagnosed more recently, born in Sweden, with longer education years, higher personal disposable income and less comorbidity, thus suggesting that these patients might survive long enough to receive the vaccination. To control this bias, the matching strategy was used to reduce the confounding effect from those factors. Consistently, we also observed the slightly larger proportion of early stage (I and II) breast cancer in vaccinated patients, so a sensitivity analysis only including patients with early stage breast cancer was performed. Another important prognostic factor is the treatment for breast cancer. Despite lacking detailed treatment information, we obtained the medication of hormonal therapy from the Swedish Prescribed Drug Register. No difference in the distribution of the therapy in the cohort stratified by cholera vaccination was found thus demonstrating the unlikely discrepancy of breast cancer treatment in Sweden where universal healthcare is accessible for all citizens at a minimal cost. As for the indication bias, the reasons to have cholera vaccine after breast cancer diagnosis were unknown, so we could not largely rule it out. However, we tried to investigate it by checking the survival in breast cancer patients with post-diagnostic antimalarial vaccination as those individuals represented a group similar to those with cholera vaccination who were able to travel abroad.

The mechanism behind the association is not clear yet, but some in vitro and in vivo studies have shown some evidence of antitumour effect of cholera toxin. Suppression of cell proliferation either by inhibiting growth factor signals or by triggering apoptosis was observed in several cancer cell lines treated with cholera toxin, including bladder,^19^ ovarian,^20^ breast,^5^ lung^5^ and pancreatic cancers,^21^ hepatocellular carcinoma and glioma.^22^ Cho-Chung et al. reported growth arrest of 7,12-dimethylbenz(a)anthracene-induced mammary carcinoma in rats treated with a daily injection of cholera toxin, and the tumours shrank 85% in 4–5 weeks.^23^ Similar results were found in human breast cancer cells (MCF-7).^23^ Growth inhibitions both in vivo and in vitro were dose dependent and correlated with increases of cyclic adenosine 3’:5’-monophosphate (cAMP) content and type II cAMP-dependent protein kinase activity as well as a decrease of oestrogen-binding activity.^23^ In addition, acetylation of P53 protein was observed in cultured MCF-7 cells treated with CTB subunit by upregulating the expression of P300, an enzyme that acetylates histones, and consequently it induced apoptosis.^5^ Antitumour effects of cholera toxin may partly be attributed to its immunomodulatory properties. It is considered to be a promising drug in treatment of autoimmune and allergic diseases.^24^ Recombinant CTB subunit could promote dendritic cell maturation presenting with upregulated expression of major histocompatibility complex class II and B7-2 on dendritic cell and enhanced secretion of interleukin (IL)-12 from dendritic cell, which is important for T cell stimulation and further antitumour immunity.^6^ Suppression of carcinogenesis in a mouse model of inflammation-driven colon cancer was observed by the oral administration of cholera toxin. This finding was accompanied with the downregulated neutrophils and upregulated regulatory T cells, IL-10 and tumour necrosis factor α in the colonic mucosa.^10^ This study indicated that gut microbiota antigenic stimuli may affect the immune system and further cancer development. As for breast cancer, the correlation between gut microbiota and mammary tumorigenesis can explain the role of immunity in our finding to some extent.^25^ Interestingly, immunomodulatory property was not only found in cholera vaccine but also seasonal influenza vaccines. Intratumoural injection of the seasonal flu shot could reduce tumour growth by increasing antitumour CD8+ T cells and decreasing regulatory B cells within the tumour. In addition, lung cancer patients with influenza infections had lower cancer-specific mortality.^26^ This further supported the possibility of protective effect of cholera vaccination in our study. However, we acknowledged that some undetected variables such as smoking, physical activity, body mass index and diet can also confound the current association although consideration of other socioeconomic factors like disposable income, educational level and place of residence could adjust them somewhat as they are correlated to each other.^27–29^ Other observational studies and clinical trials are needed to validate the association.

The strengths and limitations of the study need to be addressed. Use of Swedish nationwide registry data provided adequate sample size and, consequently, enough statistical power to detect the difference in survival between vaccinated and unvaccinated patients. It also enabled us to avoid information bias by providing an accurate record on the cancer identification and drug administration. By linking several Swedish registers, a facet of demographic, clinical and socioeconomic factors, which may affect breast cancer survival, could be considered for adjustment. Some other health-related indicators such as smoking, physical activity, body mass index and diet were not available in our study, which may affect our findings. However, consideration of other socioeconomic factors like disposable income, educational level and place of residence can adjust them on some level. Multiple sensitivity analyses were done, which strengthened the robustness of the results. Notably, analysis of the association between antimalarial medication and breast cancer survival was performed to avoid the indication bias, given the fact that vaccinated patients might be healthier and associated with better socioeconomic status. Application of matching design improved the comparability between groups and, in addition, helped avoid confounding. However, the protective effect of cholera vaccine was only observed in the matched patients who presented with specific characteristics, for example, largely with early clinical stage and hormonal therapy (Table 1). Studies among patients with late-stage breast cancer are needed. In addition, information on hormonal receptor status is required to investigate whether the effect is subtype specific. We were unable to analyse the dose–response effect as the variation of the patients with vaccination was very small. Further studies are required to generalise the results to the other population and to explore the dose–response relationship between cholera vaccination and breast cancer survival.

## Conclusions

Based on this nationwide study, we found that post-diagnostic use of cholera vaccine in breast cancer patients was associated with better overall and disease-specific survival. This association was still significant after considering competing risks and 1-year lag of exposure. This study suggests that cholera vaccine may be a good candidate for drug repurposing for breast cancer. However, our results should be interpreted carefully as some other undetected factors such as physical activity and dietary habits may have masked the current association despite our stringent analyses. Further studies are required to validate our finding in other populations and to explore the mechanisms behind the observed associations.

## Supplementary information

Supplementary table 1

## Data Availability

The use of these data is governed by an agreement with the Swedish National Board of Health and Welfare with J.S., which does not allow redistribution of original data. Anyone who is interested in the data set should contact the Swedish National Board of Health and Welfare and apply for the access to the data set (https://www.socialstyrelsen.se/statistics). If anyone gets the approval, they can get access to the database in the same manner as the authors. The project database is located at Center for Primary Health Care in Malmö, Sweden.
