# Supplementary material for: Association of post-diagnostic use of cholera vaccine with survival outcome in breast cancer patients
Source: Br J Cancer. 2020 Oct 7;124(2):506–12. doi: 10.1038/s41416-020-01108-9 (PMC7852596; doi:10.1038/s41416-020-01108-9)
Supplement: Supplementary file 1 — Supplementary table 1 [file 41416_2020_1108_MOESM1_ESM.docx]

S Table 1 Characteristics of breast cancer patients diagnosed from 2005 to 2014 stratified by cholera vaccination in Swedish Cancer Registry

| Characteristics | | No use | | Cholera vaccine use | | P-value |
| --- | --- | --- | --- | --- | --- | --- |
|  |  | N | % | N | % |  |
| Age at diagnosis (years old) | ≤ 65 | 27069 | 52.2 | 494 | 80.1 | < 0.0001 |
|  | > 65 | 24768 | 47.7 | 123 | 19.9 |  |
| Year of diagnosis | 2005-2010 | 23880 | 46.0 | 488 | 79.1 | < 0.0001 |
|  | 2011-2014 | 27957 | 53.9 | 129 | 20.9 |  |
| Birth country | Sweden | 44541 | 85.9 | 559 | 90.6 | 0.0014 |
|  | Other European countries | 4995 | 9.6 | 46 | 7.5 |  |
|  | Non-European countries | 2301 | 4.4 | 12 | 1.9 |  |
| Education level, years | 1-9 | 14069 | 27.1 | 52 | 8.4 | < 0.0001 |
|  | 10-11 | 20270 | 39.1 | 229 | 37.1 |  |
|  | ≥ 12 | 16936 | 32.6 | 336 | 54.5 |  |
|  | Missing | 562 | 1.1 | 0 | 0.0 |  |
| Disposable income | Lowest | 13160 | 25.4 | 78 | 12.6 | < 0.0001 |
|  | Middle-low | 13164 | 25.4 | 121 | 19.6 |  |
|  | Middle-high | 12643 | 24.4 | 164 | 26.6 |  |
|  | Highest | 12099 | 23.3 | 251 | 40.7 |  |
|  | Missing value | 771 | 1.5 | 3 | 0.5 |  |
| Place of residence | Big cites | 25917 | 50.0 | 329 | 53.3 | < 0.0001 |
|  | Southern Sweden | 15756 | 30.4 | 182 | 29.5 |  |
|  | Northern Sweden | 8981 | 17.3 | 106 | 17.2 |  |
|  | Missing | 1183 | 2.3 | 0 | 0.0 |  |
| Comorbidity* | No | 40519 | 78.1 | 526 | 85.3 | < 0.0001 |
|  | Yes | 11318 | 21.8 | 91 | 14.7 |  |
| Aspirin use | No | 40014 | 77.1 | 523 | 84.8 | < 0.0001 |
|  | Yes | 11823 | 22.8 | 94 | 15.2 |  |
| Clinical stage | Ⅰ | 19622 | 37.8 | 241 | 39.1 | 0.0003** |
|  | Ⅱ | 28950 | 55.8 | 363 | 58.8 |  |
|  | Ⅲ | 1868 | 3.6 | 9 | 1.5 |  |
|  | Ⅳ | 1397 | 2.7 | 4 | 0.6 |  |
| Hormone therapy *** | No | 12315 | 23.7 | 149 | 24.1 | 0.8201 |
|  | Yes | 39522 | 76.2 | 468 | 75.9 |  |
| Total |  | 51837 | 100 | 617 | 100 |  |

* Diseases for the calculation of Charlson Comorbidity Index were considered: myocardial infarction, congestive heart failure, peripheral vascular disease, cerebrovascular disease, dementia, chronic pulmonary disease, rheumatic disease, peptic ulcer disease, mild liver disease, diabetes, hemiplegia or paraplegia, renal disease, any malignancy (including lymphoma and leukemia, except malignant neoplasm of skin), moderate-to-severe liver disease, metastatic solid tumor and AIDS/HIV (15).

** Fisher Exact test was performed.

***Hormone therapy included anti-estrogens (ATC, L02BA), aromatase inhibitors (L02BG) and gonadotropin-releasing hormone analogues (L02AE).
